# The Frequency of Granulocytes with Spontaneous Somatic Mutations: A Wide Distribution in a Normal Human Population

**DOI:** 10.1371/journal.pone.0054046

**Published:** 2013-01-14

**Authors:** Tommaso Rondelli, Margherita Berardi, Benedetta Peruzzi, Luca Boni, Roberto Caporale, Piero Dolara, Rosario Notaro, Lucio Luzzatto

**Affiliations:** 1 Laboratory of Cancer Genetics and Gene Transfer, Core Research Laboratory (CRL) - Istituto Toscano Tumori (ITT), Firenze, Italy; 2 Clinical Trials Coordinating Center, CRL-ITT, Firenze, Italy; 3 Flow Cytometry Unit, Careggi Hospital, Firenze, Italy; 4 Department of Pharmacology, University of Florence, Firenze, Italy; 5 Istituto Toscano Tumori, Firenze, Italy; University of Bristol, United Kingdom

## Abstract

Germ-line mutation rate has been regarded classically as a fundamental biological parameter, as it affects the prevalence of genetic disorders and the rate of evolution. Somatic mutation rate is also an important biological parameter, as it may influence the development and/or the course of acquired diseases, particularly of cancer. Estimates of this parameter have been previously obtained in few instances from dermal fibroblasts and lymphoblastoid cells. However, the methodology required has been laborious and did not lend itself to the analysis of large numbers of samples. We have previously shown that the X-linked gene PIG-A, since its product is required for glycosyl-phosphatidylinositol-anchored proteins to become surface bound, is a good sentinel gene for studying somatic mutations. We now show that by this approach we can accurately measure the proportion of PIG-A mutant peripheral blood granulocytes, which we call mutant frequency, ƒ. We found that the results are reproducible, with a variation coefficient (CV) of 45%. Repeat samples from 32 subjects also had a CV of 44%, indicating that ƒ is a relatively stable individual characteristic. From a study of 142 normal subjects we found that log ƒ is a normally distributed variable; ƒ variability spans a 80-fold range, from less than 1×10^−6^ to 37.5×10^−6^, with a median of 4.9×10^−6^. Unlike other techniques commonly employed in population studies, such as comet assay, this method can detect any kind of mutation, including point mutation, as long as it causes functional inactivation of PIG-A gene. Since the test is rapid and requires only a small sample of peripheral blood, this methodology will lend itself to investigating genetic factors that underlie the variation in the somatic mutation rate, as well as environmental factors that may affect it. It will be also possible to test whether ƒ is a determinant of the risk of cancer.

## Introduction

Somatic mutations are known mostly as a source of disease [1], [2], and they are often associated with exposure to mutagens [3]. However, somatic mutations also occur spontaneously, as a result of the fact that DNA replication is highly faithful but cannot be perfect [4]; therefore the rate of somatic mutation, µ, is a measure of how far or how near to perfection DNA replication can be kept. In human cells µ is estimated to be of the order of 10^−7^ per gene per cell division [5]–[7]. We can presume that many inherited factors will determine µ, including all the genes that are involved in the proof-reading of the DNA replication process and in DNA repair [8]; at the same time, exogenous mutagens will, by definition, increase µ. However, there is hardly any information about the extent of variation in the values of µ, whether in exposed or in unexposed individuals.

Recently we have developed a method to measure µ in lymphoblastoid cell lines that can be obtained from a peripheral blood sample [9], and we have an estimate of the normal range [6]. The method is based on using as sentinel gene *PIG-A*, which encodes a component of an enzyme required for the biosynthesis of the glicosylphosphatidylinositol (GPI) molecule, which serves as anchor for many surface proteins. Since *PIG-A* is X-linked, a single inactivating mutation in this gene will cause deficiency of all GPI-linked proteins, and this can be conveniently demonstrated on individual cells by flow cytometry. The same approach has been applied to red cells, granulocytes and lymphocytes of humans and of laboratory animals [10], [11].

Compared to other methods that have been previously employed for the measurement of µ [5], the *PIG-A*-based methodology is more convenient [9], but it is still laborious, mainly because it requires obtaining a lymphoblastoid cell line from each subject to be tested: therefore it seems important to explore even simpler techniques. It is not possible, by definition, to measure µ in non-dividing cells: however, it is easy to measure the frequency *(ƒ)* of cells with a specific phenotype that must have arisen through a mutation in one of their precursors. In this paper we show in detail how *ƒ* values can be accurately measured within hours on granulocytes from a small peripheral blood sample; we have further determined reproducibility and normal range of *ƒ* (granulocytes) in 142 healthy subjects.

## Materials and Methods

### Subjects

Peripheral blood samples were collected in EDTA from 142 healthy donors (50 women and 92 males) whose age ranged between 23 and 63 years. Previously 60 found blood samples were used to set up the procedure. Signed informed consent was obtained according to an IRB approved protocol. Blood samples were stored at room temperature and promptly processed within 2 hours from collection.

### Purification of Peripheral Blood Granulocytes

Granulocytes were prepared from about 7 ml of freshly collected (see above) blood samples by a one-step double density centrifugation method [12] that has been modified in the aim of obtaining highly purified granulocytes, as follows.

**Figure 1:**
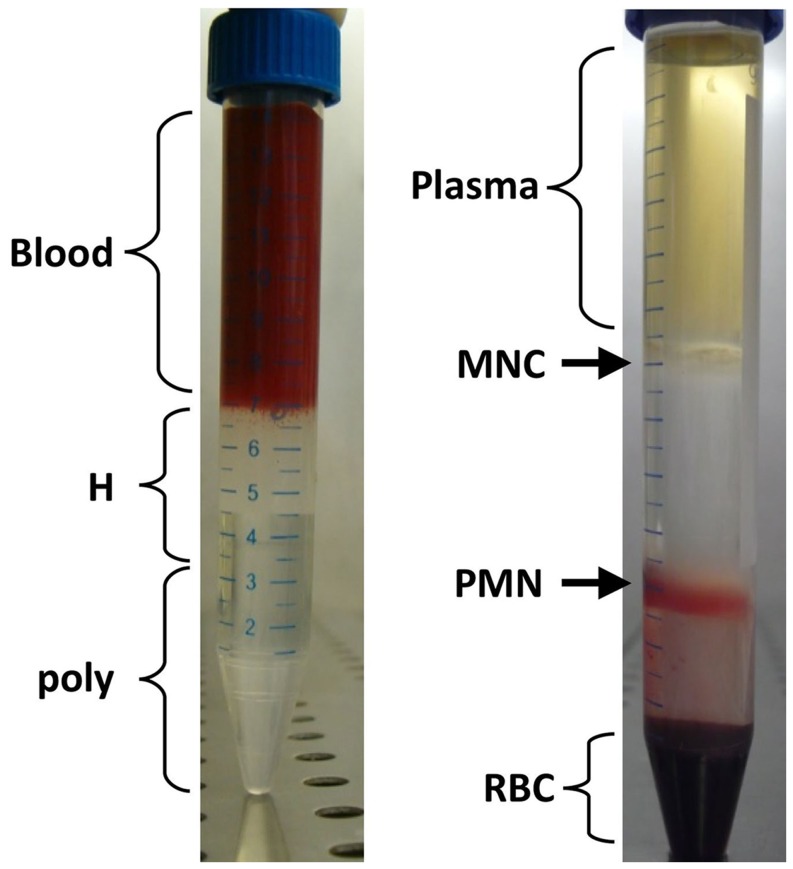
*Purification of peripheral blood Granulocytes*. Representative example of preparation of granulocytes by a one-step double density centrifugation method [12]. In the left-hand panel, a whole blood sample diluted 1∶3 in PBS is layered on a double density Ficoll gradient: poly (density 1113 g/L) and H (density 1077 g/L). In the right-hand panel one sees that, after centrifugation (500 g for 35 minutes at 22°C), erythrocytes (RBC), granulocytes (PMN), mononuclear cells (MNC) and plasma fractions are clearly separated. The relatively few erythrocytes contaminating the granulocyte fraction are subsequently removed by gentle osmotic lysis.

Each whole blood sample was diluted 1∶3 with sterile PBS at room temperature to a final volume of about 21 ml (blood: 7 ml; PBS: 14 ml). A double density gradient was prepared in 15 ml polypropylene tubes (Greiner bio-one, N.188271) by carefully layering 3.5 ml of Ficoll with density 1077 g/L (Lympholyte H, CL5020, Cederlane) over 3.5 ml of Ficoll with density 1113 g/L (Lympholyte poly, CL5070, Cederlane). Onto the Ficoll about 7 ml of PBS-diluted blood was then carefully layered. After centrifugation (500 g for 35 minutes at 22°C, see Fig. 1) granulocytes were found at the 1077/1113 interphase (lower ring); mononuclear cells were found at the plasma/1077 interphase (upper ring); erythrocytes were at the bottom. In some cases the lower ring showed some red cells contamination, but this did not affect subsequent results. The lower ring was collected by gentle pipetting, and the resulting granulocyte suspension ranged in volume from 2 to 6 ml. In order to eliminate Ficoll (which might interfere with the next step), the suspension was diluted by adding 10 ml of PBS per each ml of suspension. After spinning at 300 g for 5 min the supernatant was removed and the pellet was re-suspended in 45 ml of a solution of NH_4_Cl 0.157M - KHCO_3_ 0.01M - EDTA 100 mM and left at 4°C for 10 min. This lysed any remaining red cells; the purified granulocytes were now pelleted by spinning again at 300 g for 5 min at 4°C. After removing the supernatant, the granulocytes were resuspended in 4 ml of PBS, a cell count was performed, and an aliquot containing 5×10^6^ granulocytes was transferred to a polystyrene tube (BD Bioscience).

### Staining of Granulocytes

The 5×10^6^ granulocytes were pelleted (300 g for 5 min at 4°C), resuspended in 150**µl of PBS, and then stained at 4°C with a mixture of 5 antibodies as follows: three PE-coniugated antibodies, each one of which recognizes one of three GPI-linked proteins (PE mouse anti human CD59 (0,5**µl/10^6^ cell; AbD Serotec, clone MEM-43), PE mouse anti-human CD55 (1**µl/10^6^ cell; BD Pharmigen, clone IA10) and PE mouse anti-human CD24 (5**µl/10^6^ cell; BD Pharmigen, clone ML5); with APC mouse anti-human CD45 (1**µl/10^6^ cell; BD Pharmigen, clone HI30) and with FITC mouse anti-human CD11b (5**µl/10^6^ cell; Milteni Biotech, clone M1/70.15.11.5). The incubation was done for 40 min at 4°C.

### Flow Cytometry Analysis

**Figure 2:**
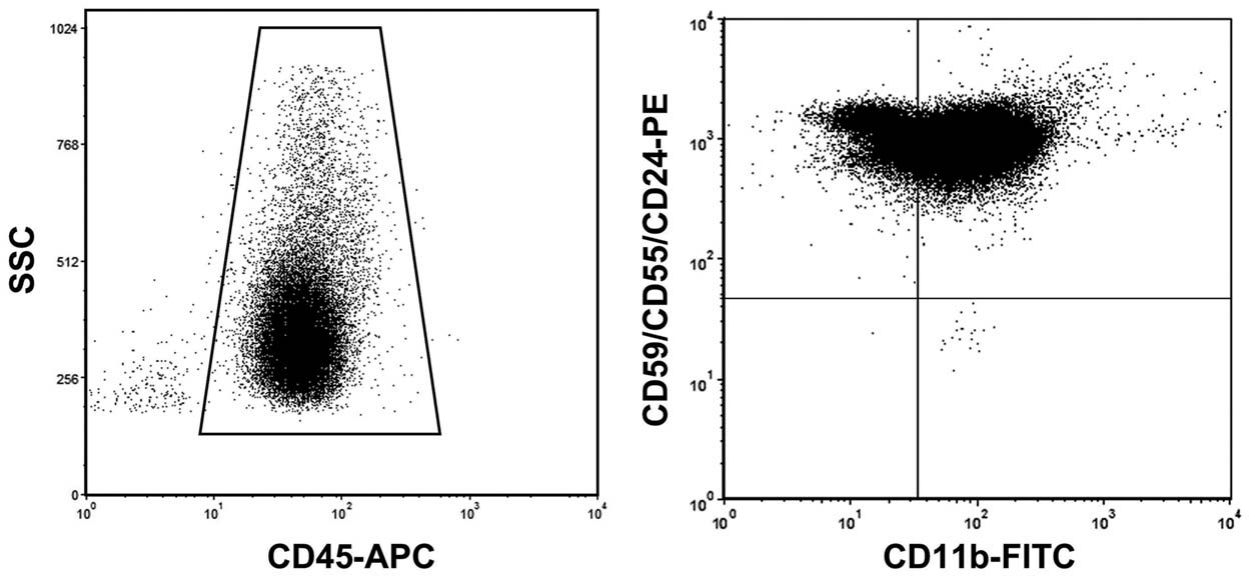
*Counting granulocytes that have a PIGA-inactivating mutation*. The left-hand panel shows the gating strategy, based on SSC and CD45, used to eliminate virtually all cells that are not granulocytes. The right-hand panel shows that granulocytes are positively identified as CD11b-positive; at the same time, GPI-negative granulocytes are identified (lower right quadrant) as cells that (while being among the 95% of cells with higher CD11b fluorescence) have a GPI-linked-PE fluorescence intensity lower than 4% of the geometric mean of the fluorescence values of all events. Mutant frequency, *ƒ*, was calculated as the number of negative events, as just defined, per million cells.

Cells were analyzed on BD FACSCanto II using FACS Diva Software. Live granulocytes were identified by physical parameters (SSC *vs*. FSC), as well as through two non-GPI-anchored membrane proteins: CD45 protein (a ‘pan-leukocyte’ marker commonly used to differentiate leucocytes *versus* any other blood cells), and CD11b, which is granulocyte-specific (Fig. 2). This combination of physical parameters with specific surface proteins is highly reliable for the identification of granulocyte events. In addition, since the identification of mutant granulocytes is based on *PIG-A* inactivation, to make the analysis highly robust we used three (rather than just one) GPI-anchored proteins: when a granulocyte tests negative for all 3 we can be fully confident that this is due to a *PIG-A* mutation, and not to a mutation in a structural gene for any of those proteins or to any other spurious event.

In the interest of accuracy the acquisition rate was set up to less than 2500 events per second. Since GPI-negative cells are rare events, in order to obtain an accurate estimate of their numbers we collected at least 1.3 million events with gating for physical characteristics and CD45 staining. Finally, in the aim to safeguard against damaged cells we have excluded from the count of granulocytes the events with the 5% lowest fluorescence for CD11b, characteristic marker of granulocytes.

### Methodology for Determining ƒ

The technique we have used is very similar to that previously described [6], [13]. This is based on a direct count, by flow cytometry analysis, of the number of granulocytes (defined as described above) that are markedly deficient in GPI-anchored proteins because of a *PIG-A* gene mutation (see Fig. 2). We have regarded as GPI-negative (*i.e.* mutant) cells all events that showed a GPI-linked-PE fluorescence intensity lower than 4% of the geometric mean of the fluorescence values of all events [9]. The threshold of 4% was based on cases that showed a clearly bimodal distribution of fluorescence (see Fig. 2B). Mutant frequency, *ƒ*, was calculated as the number of negative events, as just defined, per million cells.

### Spiked Controls

**Figure 3:**
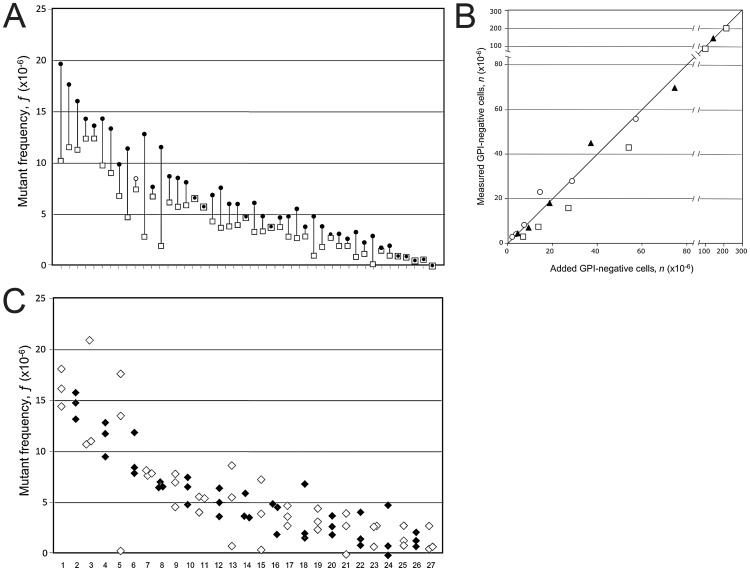
*Technical reproducibility and intra-individual variation of ƒ*. (A) In this plot we show ƒ values of 45 split samples (see Methods). For each sample the higher value is a filled circle and the lower value of ƒ is an empty square. (B) Spiked control. The X axis shows the number of the added PNH (GPI-negative) granulocytes to a control sample; the Y axis shows the number of GPI-negative granulocytes measured (after the subtraction of the ƒ value measured in the control sample). Square, diamond and circle indicate different samples. The line indicates the theoretical identity between added and measured GPI-negative cells. See “Materials and Methods” for details. (C) In this plot we show ƒ values for 27 subjects who have been tested at 3 different times. Each diamond represents one measurement of ƒ. White and black diamonds alternate in the interest of clarity. In panels A and C the data have been arranged in order of decreasing mean ƒ values, again in the interest of clarity.

Granulocytes from PNH patients (>95% GPI-negative) were suspended in buffered saline at a concentration between 2 and 6×10^4^ per ml. The suspension was first diluted 1/100; and from this dilution further two-fold dilutions were made serially to the final dilutions of 1/200, 1/400, 1/800, 1/1600, 1/3200. One ml of each of the resulting PNH granulocyte suspensions was added to a tube containing to 3×10^6^ granulocytes from a healthy control. We then measured the frequency of GPI-negative granulocytes in the unspiked control sample: this was our background. We then measured the frequency of GPI-negative granulocytes in the sample spiked with the undiluted PNH granulocytes in order to obtain a precise count of added GPI-negative granulocytes. From these two measured values we calculated the expected numbers of GPI-negative granulocytes added with the various diluted samples. These values were compared with observed values in the spiked samples. After subtracting background, we compared the two sets of values (see Fig. 3B).

### Statistical Procedures

We have performed more than 300 determinations and in about 7% of cases we have not found any mutant granulocytes out of 1.3×10^6^ cells analyzed: in these cases we have to say that *ƒ* is below the limit of detection by our method. In principle, it would be possible to overcome this problem by increasing (say by ten-fold) the number of analyzed cells, but this would not be practical. In order to estimate and to compare the distribution of *ƒ* without censoring the values below the limit of detection, referred to hereinafter for brevity as 0 (zero) values, we have used either of two available replacement methods, (1) Each 0 value was replaced by the reciprocal of the number of cells counted (*e.g.* 1/1.3×10^6^ = 0.77×10^−6^) divided by root square of 2 as reported by Hornung and Reed [14]. (2) Imputation by maximum likelihood estimation [15]: details of this are given in the “Results” section.

The reproducibility of ƒ was determined by dividing samples in two aliquots that were then processed separately. The precision error and its 95% confidence interval (CI) for split samples were estimated as described by Gluer [16]. The intra-individual variability has been estimated in terms of precision error calculated as standard errors of the estimate of changes in repeat samples [16]. In addition, the coefficient of variation (CV) has been estimated for both split samples and for repeat samples.

The comparison of distributions of values of ƒ observed in different series has been carried out by the Wilcoxon test for unpaired data. Normality of distributions has been tested by the Kolmogorov-Smirnov test. Differences between the average values of ƒ observed in different subgroups of subjects were tested by one-way analysis of variance (ANOVA). The correlation between the values of ƒ and the absolute granulocyte counts was assessed according to Pearson.

All statistical analyses have been done with SAS System 9.2 and R statistical packages.

## Results

### Yield and Purity of Granulocytes

The granulocyte separation procedure based on a double density gradient [12] followed by RBC lysis has enabled us to recover about 81±19% of granulocytes from each fresh blood sample (*n* = 64). The contamination by cells other than granulocytes was less than 3%. Thus, we have been able to stain, from virtually any blood sample, at least 5×10^6^ granulocytes, as required in order to collect about 1.3×10^6^ events in our flow cytometry analysis.

### Measurement of ƒ and Reproducibility

In order to assess the reproducibility of our assay, 87 blood samples were split in two tubes, which were then independently processed, stained and analyzed by flow cytometry. Since in 13 cases one or both measurements yielded a 0, in order to obtain estimates of mean and variance we have replaced the 0 values according to Hornung & Reed [14] (see under Methods); whereupon the estimate of the precision error of *ƒ* on split samples was 2.83 (CI 95%: 2.46–3.32); the CV was 44.7% (Fig. 3A). There was no association between precision error and the value of ƒ (P = 0.750, anova F test for trend).

As a further test of the reliability of our method we carried out a series of internal control tests, by spiking normal samples with known numbers of GPI-negative granulocytes from PNH patients. We found an excellent correlation between the number of GPI-negative cells added and that of GPI-negative cells measured (Fig. 3B: the values of *r^2^* range from 0.971 to 0.994; P≤0.0003).

### Variability of ƒ in Individual Subjects

From 32 volunteers we have obtained three repeat samples, with time intervals from 15 to 200 days between samples. It is seen (Fig. 3C) that the ƒ values are rather similar in the repeat samples from each person, by comparison to the technical reproducibility of the ƒ measurement. Zero values (6 cases) were again replaced according to Hornung & Reed [14]. The mean of the 3 measurements was 5.7×10^−6^, the intra-individual variability (precision error) was 2.54×10^6^ (CI 95%: 2.04–3.36); and the CV was 44.3%, almost identical to the CV we had established for split samples (44.7%: see above). This means that in each person the frequency *ƒ* of mutant granulocytes is relatively stable; and the variation of *ƒ* observed in time in that person can be attributed essentially to the precision error of our assay.

### The Normal Range of ƒ

**Figure 4:**
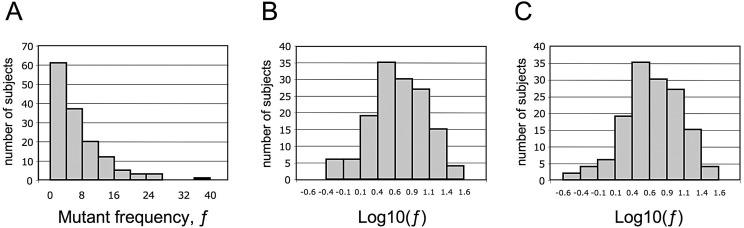
Histogram distribution of ƒ in a population of 142 healthy individuals. A. On a linear scale ƒ has a highly asymmetric truncated distribution. B. On a log scale ƒ has a truncated distribution, where the truncation clearly results from the ‘zero’ values. C. After replacement of the zero values with values obtained by maximum likelihood estimation (see text) the distribution of ƒ on a log scale is not significantly different from a normal distribution.

Having established the reproducibility and the intra-individual stability of *ƒ,* we proceeded to establishing its normal range. We have measured *ƒ* in an additional set of 110 healthy subjects. Since in this set the distribution of *ƒ* values was not significantly different (Wilcoxon two-sample test: *P*>0.603) from that observed in the previous set of 32 subjects from whom we had obtained repeat samples, we pooled the data from all 142 subjects (Fig. S1). In this series ƒ values ranged between less than 1 up to 37.5×10^−6^. A histogram of these data (Fig. 4A) shows a highly asymmetric truncated distribution (which does not remotely look like a normal distribution: *P*<0.001). After log transformation the histogram resembles a truncated normal distribution (*P*<0.15, Fig. 4B), where the truncation clearly results from the 0 values. In order to obtain a more accurate estimate of mean and variance of this distribution we have resorted to a maximum likelihood estimation method reported by Hald [15], whereby non-zero values are imputed to those samples in which mutant cells were below the limit of detection, in a manner that optimizes the symmetry of the distribution (Fig. 4C). The normal distribution of log-transformed *ƒ* thus obtained has a median of 0.69, a mean of 0.67 and a standard deviation of 0.41. After imputation, the median value of *ƒ* on the original scale is 4.9×10^−6^, with 95% of values below 19.8×10^−6^, and 99% of values below 26.7×10^−6^.

When we stratified our subjects according to gender, we found no significant differences in the average values of log-transformed *ƒ* (P = 0.447, anova F test). When we stratified our subjects according to age there we also found no significant differrences (P = 0.757, anova F test). Moreover, no correlation was found between the values of log-transformed *ƒ* and absolute granulocyte counts (Pearson’s *r* = 0.11, P = 0.299). Similar results were obtained when instead of log transformed values we used the original *ƒ* values.

## Discussion

Somatic mutations have first become prominent in human biology with the discovery that they have a major physiological role in immunity, but we learnt promptly that hyper-mutation was a unique characteristic of few special genes confined to specific cell lineages, the B and T lymphoid cells [17]. Otherwise, somatic mutations have long been regarded as an ominous phrase associated with cancer. However, considering that DNA replication cannot be perfect, spontaneous somatic mutations must be relatively common: and it has been estimated that for any individual gene mutant cells must be present in the body [18].

In keeping with this notion, we have previously shown that granulocytes with inactivating mutations of the GPI biosynthetic gene *PIG-A* can be demonstrated in peripheral blood granulocytes of normal persons [13]. We have also discussed [9] why this is a particularly useful gene as a sentinel of somatic mutations: briefly, there are three reasons. (i) *PIG-A* is X-linked and subject to X inactivation [19], [20]: therefore a single inactivating mutation, whether in a male or in a female cell, will give a distinct cellular phenotype. (ii) Because the GPI anchor is required to tether proteins to the cell membrane [21], the phenotype is expressed on the cell surface and thus easily accessible to flow cytometry. (iii) The reliability is high, because the same *PIG-A* mutation produces deficiency of all GPI-linked protein, and we can test each cell for more than one. Most of the known mutations that inactivate *PIG-A* in PNH patients are point mutations or small indels [22], but larger deletions have also been seen, and of course other genetic rearrangement are potentially inactivating [6], [13], [22]. In principle, epigenetic changes might also silence *PIG-A*: in fact, possible epigenetic silencing must be regarded as part of the intrinsic background of any method based on counting mutant cells on the basis of a deficient phenotype. However, with respect to *PIG-A* if such epigenetic events (such as promoter hyper-methylation) do occur they must be rare: indeed, in previous experiments whenever we have sequenced *PIG-A* from even very few GPI-negative cells from healthy individuals we have always found an inactivating mutation [6], [13].

There are several reasons why it may prove important to have a measure of the frequency of somatic mutations in humans. First, the individual variability of the rate of somatic mutations in human populations was not known hitherto. Second, a test that is based on inactivating mutations of a specific gene would be a useful sensor of exposure to environmental mutagens, and it would complement other popular genotoxicity tests, such as the micronuclei [23] and comet [24] assays, which detect other kinds of DNA damage such as single-strand and double-strand breaks. Third, a test for the intrinsic tendency of each individual to make somatic mutations that is sufficiently simple to perform might prove a new way to explore individual susceptibility to develop tumors.

It has not been proven as yet that the frequency *ƒ* of mutant peripheral blood granulocytes is proportional to the intrinsic somatic mutation rate µ. Since granulocytes are end cells of myeloid differentiation the number of PIG-A mutant granulocytes in the peripheral blood will depend on the stage at which the PIG-A mutation has taken place: if it has taken place at an early stage, say in a CFU-GEMM cell, we will find in the blood many more GPI deficient granulocytes than if the mutation has taken place at the last cell division of a maturing myelocyte. Until proven otherwise, we presume that, in first approximation, *ƒ* results from an even mix of early and late mutational events. We must also consider that µ may not be the same for all types of cells within the same person, for several reasons. (i) In maturing cells belonging to the same lineage m may vary at different stages of maturation, for instance because the efficiency of DNA repair mechanisms may have changed: at the moment this is not known. (ii) µ may not be the same in different types of somatic cells, for instance in granulocytes versus lymphocytes versus epithelial cells: however, it seems reasonable to surmise that the values of µ in various cell lineages will be proportional to each other in any individual person. (iii) The mutation rate may be affected by the environment. Therefore, when we measure µ in lymphoblastoid cell lines under standardized culture conditions the values of µ we obtain must be very near to the intrinsic somatic mutation rate; whereas when we measure *ƒ* we are automatically including also possible effects that the environment may have had in vivo on granulocytopoiesis in that particular individual at that particular time. In spite of these limitations we suggest that, until proven otherwise, in general *ƒ* may be a reasonably acceptable surrogate measure of µ.

The data presented in this paper show that the reproducibility of the test is good, with a coefficient of variation is 44,7%. This result has been obtained through rigorous standardization of technique, and particularly by carrying out the test within hours of blood collection: although we have found, in a limited number of experiments (data not shown) that the results after overnight storage of samples are not significantly different. From the biological point of view, a very significant finding was that intra-individual variation, 44,3%, on repeat samples was practically the same as intra-sample variation. Therefore ƒ is proven to be a rather robust parameter associated with the individual, suggesting that it reflects quantitatively the intrinsic tendency of that individual to generate somatic mutations.

In contrast to a limited intra-individual variation, inter-individual variation was considerable; and after log transformation the ƒ values are normally distributed. The average of the ƒ values imputed to the samples that had no detectable mutant cells is 0.48×10^−6^, and since the highest value is 37.5×10^−6^, the span of ƒ values is about 80-fold.

Interestingly, a very similar range of ƒ values (except for two outliers) has been reported recently by Dobrovolsky *et al.* [25] in human red cells from normal subjects. We have chosen to assess ƒ in granulocytes rather than in red cells, despite the fact that the task is more laborious, because there is abundant evidence from the study of PNH patients that GPI-deficient red cells have a shortened life span due to complement–mediated lysis *in vivo* (see Dacie [26], Rosse [27], Luzzatto & Notaro [28]).

The biological basis for inter-individual variation is not yet known. Unlike patients with Fanconi anemia or with ataxia-telangectasia, in whom we have previously shown that the somatic mutation rate is markedly increased [6], the subjects tested here were all normal healthy subjects. However, it is possible that polymorphic variants of genes such as those of the Fanconi complex (which are no less that a dozen [29], [30]) or of ataxia-telangectasia, which may never cause overt disease, might affect the mutation rate. The same could be true, in principle, for any gene involved in proofreading, in mismatch repair, or in other forms of DNA repair. This method may also prove useful for quantitative testing of the mutagenic effect of therapeutic protocols. Having established a normal range for *ƒ,* we are now in a position to ask whether, within the normal range, higher values of ƒ signify an increased risk of cancer. We don’t yet have evidence in this respect; but we must consider that, when compared to other inherited risk factors, mutation rate is rather different. Indeed, cancer results from a multi-step process for which several somatic mutations are required. Although the number *n* of mutations is not known in most cases, and it is likely to be different in different tumors [31], the probability of accumulating the same *n* mutations for an individual who has a mutation rate double the average, will be 2*^n^* greater than average (meaning a factor of 32 for a tumor for which *n* = 5). Thus, the impact of relatively small differences in the rate of somatic mutations would be considerably amplified, and it seems now possible to test this hypothesis.

## Supporting Information

Figure S1 **Distribution of ƒ in a population of 142 healthy individuals.** In this scattergram each dot represent the ƒ value of one healthy individual. When more than one measurement was available the average ƒ is shown.
(PDF)
