# Supplementary material for: The Frequency of Granulocytes with Spontaneous Somatic Mutations: A Wide Distribution in a Normal Human Population
Source: PLoS One. 2013 Jan 14;8(1):e54046. doi: 10.1371/journal.pone.0054046 (PMC3544671; doi:10.1371/journal.pone.0054046)
Supplement: Figure S1 — Distribution of ƒ in a population of 142 healthy individuals. In this scattergram each dot represent the ƒ value of one healthy individual. When more than one measurement was available the average ƒ is shown. (PDF) [file pone.0054046.s001.pdf]

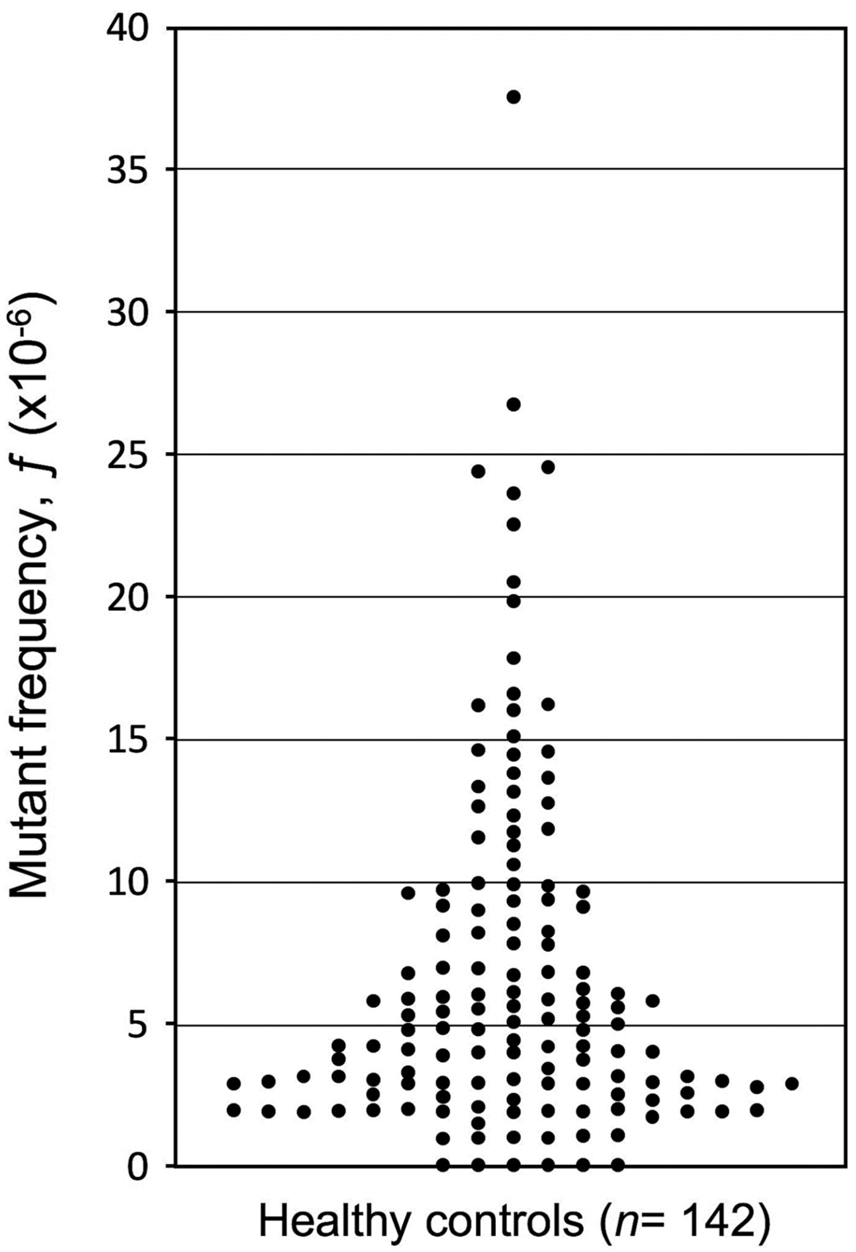

Supplement Figure 1. *Distribution of ƒ in a population of 142 healthy individuals.* In this scattergram each dot represent the ƒ value of one healthy individual. When more than one measurement was available the average ƒ is shown.
